# A Tri-Stage Wrapper-Filter Feature Selection Framework for Disease Classification

**DOI:** 10.3390/s21165571

**Published:** 2021-08-18

**Authors:** Moumita Mandal, Pawan Kumar Singh, Muhammad Fazal Ijaz, Jana Shafi, Ram Sarkar

**Affiliations:** 1 Department of Computer Science and Engineering, Jadavpur University, Kolkata 700032, India; moumita.mandal.iiit@gmail.com (M.M.); ram.sarkar@jadavpuruniversity.in (R.S.); 2 Department of Information Technology, Jadavpur University, Kolkata 700106, India; pksingh.it@jadavpuruniversity.in; 3 Department of Intelligent Mechatronics Engineering, Sejong University, Seoul 05006, Korea; 4 Department of Computer Science, College of Arts and Science, Prince Sattam bin Abdul Aziz University, Wadi Ad-Dwasir 11991, Saudi Arabia; j.jana@psau.edu.sa

**Keywords:** feature selection, filter method, wrapper method, whale optimization algorithm, arrhythmia, disease classification, cancer dataset

## Abstract

In machine learning and data science, feature selection is considered as a crucial step of data preprocessing. When we directly apply the raw data for classification or clustering purposes, sometimes we observe that the learning algorithms do not perform well. One possible reason for this is the presence of redundant, noisy, and non-informative features or attributes in the datasets. Hence, feature selection methods are used to identify the subset of relevant features that can maximize the model performance. Moreover, due to reduction in feature dimension, both training time and storage required by the model can be reduced as well. In this paper, we present a tri-stage wrapper-filter-based feature selection framework for the purpose of medical report-based disease detection. In the first stage, an ensemble was formed by four filter methods—Mutual Information, ReliefF, Chi Square, and Xvariance—and then each feature from the union set was assessed by three classification algorithms—support vector machine, naïve Bayes, and *k*-nearest neighbors—and an average accuracy was calculated. The features with higher accuracy were selected to obtain a preliminary subset of optimal features. In the second stage, Pearson correlation was used to discard highly correlated features. In these two stages, XGBoost classification algorithm was applied to obtain the most contributing features that, in turn, provide the best optimal subset. Then, in the final stage, we fed the obtained feature subset to a meta-heuristic algorithm, called whale optimization algorithm, in order to further reduce the feature set and to achieve higher accuracy. We evaluated the proposed feature selection framework on four publicly available disease datasets taken from the UCI machine learning repository, namely, arrhythmia, leukemia, DLBCL, and prostate cancer. Our obtained results confirm that the proposed method can perform better than many state-of-the-art methods and can detect important features as well. Less features ensure less medical tests for correct diagnosis, thus saving both time and cost.

## 1. Introduction

In the domain of machine learning (ML) and statistics, feature selection is treated as an important step of data preprocessing. It is used to detect the subset of relevant features or attributes that maximizes the model performance [1,2]. Apart from this, there are other benefits of applying feature selection, including shorter training time, reduced dimensionality of the original feature vector, and simplification of the models by making them easier to be interpreted by the users, thus building faster models. This, in turn, also helps to gain a better understanding of the processes described by the data by focusing only on the required subset of features. In many instances, it has been seen that datasets contain unnecessary, noisy, or redundant information and such unrequited data can be eliminated by applying feature selection methods without losing any salient data. It has been proven to be very essential in the field of medical diagnosis. With this exponential growth of investments in information technology of health service provisions and widespread collection and generation of data by medical establishments, there are increased irregularities such as large numbers of disease markers. Some of the disease markers are not helpful, while some even affect the diagnosis negatively [3].

Feature selection algorithms basically revolve around the idea of selecting the optimal subset of features from the original feature set, which will help in marking the prime features as well as will improve the ML models’ performance. The easiest way to find out the optimal one would be to select every possible permutation of the subset and select the one that maximizes the performance of the model. However, this process would be very vigorous as well as computationally expensive, and for a large dataset in particular it can take a large amount of time to keep track of every subset. Generally, feature selection approaches can broadly be classified into wrapper, filter, and embedded methods [4]. Moreover, hybrid combinations of these approaches are also used by researchers [5]. 

In the filter method, prime attributes are chosen from the properties of the datasets only without considering any classification model. It rejects the least contributing attributes, and pivotal attributes are selected on the basis of statistical significance to either classify or predict data. On the contrary, the optimal subset is chosen from the feature set on the basis of a specific ML algorithm in a wrapper method. On the basis of a particular evaluation criterion, some intelligent search approaches are followed by a wrapper method by evaluating various combinations of feature subsets. Embedded methods are quite similar to wrapper methods, with only difference being that feature selection is performed during the model training only. It helps to choose better features for that model in less time. Only one downside of embedded methods is that the feature selection occurs depending on the classifier hypothesis. However, it is very obvious that it may not give the best result if any other classifier is used, and it is also dataset-dependent. Hybrid approaches are very simple, as they use the combined power of different filter and wrapper methods to select the optimal feature set. 

As discussed above, the main objective of any feature selection method remains the same, which is to maximize the model’s predictive performance. Hybrid methods are a promising approach for an improved selection process. The major advantage of hybrid methods is that they attain the top advantages from each feature selection method, which in turn results in predicting higher accuracy and reducing the computational complexity than wrapper methods. The basic goal is to produce a better predictive model by taking advantage of the different algorithms and also overcoming their weaknesses at the same time.

Keeping the above facts in mind, a new tri-stage wrapper-filter feature selection framework is proposed in this work. Five filter methods have been used in two phases, and a wrapper method is used in the third and the final phase. An ensemble of four filter methods, namely, Mutual Information (MI), ReliefF (RFF), Chi Square (CS), and Xvariance (XV) is used in the first phase, whereas Correlation is used in the second phase. Finally, a meta-heuristic, called Whale Optimization Algorithm (WOA), is used as the wrapper method in the last phase to obtain the optimal feature subset. Union of the best-chosen features selected by the four filters individually has been formed to utilize their common strength. Then accuracy of each feature is calculated on the basis of three popular ML algorithms—support vector machine (SVM), naïve Bayes (NB) and *k*-nearest neighbors (KNN), and then mean accuracy value is taken. This step ensures if an important feature is anyhow wrongly eliminated by a particular filter method, any other filter method can include them in the set. Next, highly correlated features are rejected to confirm that redundant attributes are neglected, and at the end, WOA makes certain that only prime attributes are chosen to achieve the highest accuracy. 

The study in this paper was performed on four clinical datasets, namely, arrhythmia [6], leukemia [7,8], diffuse large b-cell lymphomas (DLBCL) [9], and prostate cancer [10]. Among these, the prostate cancer dataset consists of as many as 12,533 attributes but only 102 instances. On the other hand, the arrhythmia dataset contains only 279 attributes but 452 instances. Thus, the proposed tri-stage method was applied for datasets of any dimension—large or small—and its performance was measured. As far as medical datasets are concerned, from a medical analysis perspective, it is of immense importance to find and select only necessary attributes and completely discard the noisy and irrelevant ones. Lesser attributes mean there will be less medical tests to diagnose correctly. This would ensure a reduction in the diagnosis time with a lesser number of medical tests and treatment can be started earlier, and at the same time it will certainly aid in lessening the economic burden on the patients, thus expanding the coverage of the community.

### Contributions

The contributions of the proposed work can be highlighted as follows:

1. A tri-stage wrapper-filter-based feature selection framework was proposed for the purpose of medical report-based disease detection.

2. At the first two stages, four filter methods were applied judiciously to obtain a preliminary subset of optimal features.

3. In the next stage, the obtained feature subset was fed to a meta-heuristic, called WOA, to obtain a more optimized subset of features.

4. Three commonly used classifiers—SVM, NB, and KNN—were used to prove the non-biasness of the feature selection framework toward any particular classifier.

The structure of the paper is as follows: Section 2 is dedicated to the literature survey and motivation behind the present work whereas in Section 3, the methodology for the proposed tri-stage wrapper-filter feature selection method is discussed along with a description of the datasets. Section 4 contains a detailed discussion about the results obtained and Section 5 finally concludes the paper.

## 2. Literature Survey

As feature selection is considered to be a crucial data preprocessing part, over the years, many researchers have proposed different kinds of novel feature selection approaches on disease datasets. In this section, some of the methods applied on arrhythmia, leukemia, DLBCL, and prostate cancer datasets are briefly mentioned.

### 2.1. Arrhythmia

Heart arrhythmia [6] is a disorder that affects the rate or rhythm at which the heart beats. It is considered to be the most major sign of a heart disease and can be responsible for causing strokes or cardiac arrest. It can even damage the brain, lungs, and other vital organs if it interferes with the blood flow. Thus, it is very crucial that they are detected and treated to avoid any life-threatening situation.

Xu et al. [11] experimented with different feature selection methods and classification algorithms to increase the classification accuracy of the heart arrhythmia dataset. They achieved best accuracies by using neural networks only, deep neural networks only, Fisher discriminant ratio + deep neural network, and principal component analysis + deep neural network, and they used 10-fold cross-validation with each method to achieve 82.22%, 81.42%, 82.96%, and 75.22% accuracies, respectively, for each method. Singh et al. [12] implemented three feature selection methods, namely, symmetric uncertainty, CS, and gain ratio to choose the optimal feature set and used the classification algorithms, namely, linear SVM, random forest, and a decision tree algorithm named JRip to classify. They obtained 85.58% accuracy with a 30 feature subset using gain ratio and KNN classifier.

Sahebi et al. [13] proposed a novel wrapper-based feature selection named GeFes. They used a new operator in genetic algorithm (GA) that has increased the performance of mutation and crossover operators used in GeFes. The GeFes uses KNN classifier and an integrated nested cross-validation process. They achieved an accuracy of 99.02% with 135 selected features. Cui et al. [14] proposed a feature selection technique that combines the benefit of both minimum redundancy maximum relevance (mRMR) method and improved dragonfly algorithm (IDA), naming it hybrid improved dragonfly algorithm (HIDA). Firstly, they used mRMR to discard irrelevant features and then they introduced dynamic swarming factors and quantum local optimum and global optimum to amplify the exploitation capability of IDA. They achieved 74.77% accuracy for the arrhythmia dataset and selected an average of 169.4 features.

Kadam et al. [15] introduced a two-stage feature selection method. In the first stage, they used GA to select the optimal feature set and selected 72 features in this stage. In the final stage, they implemented SVM Ensemble with bootstrap aggregating (Bagging) for both classification and evaluation purposes. They achieved 88.72% accuracy with 92 features. Wang et al. [16] applied an oversampling technique named SMOTE to balance the dataset, and next K-part Lasso was also applied to remove the existing redundant features. In the final step, they formed a feature selection method, named RF-RFE, by combining recursive feature elimination and random forest classifier together. This method obtained an accuracy of 98.68% with a total of 89 features.

### 2.2. Leukemia

Leukemia [7,8] is the most common blood cell cancer, being found in children younger than 15 years. Two most frequent types of leukemia among children are acute lymphoblastic leukemia (ALL) and acute myeloid leukemia (AML). ALL in particular affects children aged 2–10 years, whereas AML can be found in approximately 20% of adult acute leukemia cases and has a more aggressive impact.

Wang et al. [17] developed an advanced adaptive elastic net algorithm combined with conditional mutual information, naming this algorithm AEN-CMI. They applied this method on a leukemia dataset and compared their method with many state-of-the-art methods such as SVM, Adaptive Lasso, and different variants of elastic net algorithm. They ran AEM-CMI 20 times and achieved an average accuracy of 91.05%, selecting almost 27 features. Sun et al. [18] implemented the neighborhood multi-granulation rough sets (NMRS)-based feature selection method. They integrated Lebesgue and entropy measures in the NRMS method to analyze and reduce the uncertainty measures of incomplete neighborhood decision systems. Lastly, the Fisher linear discriminant method has been applied to select important features. The KNN and C4.5 classification algorithms have been used to measure the accuracy. The method was named PDJE-AR and it has achieved an average accuracy of 87.5% with an average of 7.6 features using KNN classifier.

Khamess et al. [19] presented a hybrid feature selection technique that combines sine cosine algorithm with cuckoo search algorithm to obtain the optimal feature set. They achieved 90.88% accuracy for the leukemia dataset with an average of 27.91 features. Kilicarslan et al. [20] implemented a unique hybrid method. At first, they used RFF and a stacked autoencoder method as filter methods, and then used convolution neural network (CNN) and SVM as classifiers. They then carried out the experiment with 60–40%, 70–30%, and 80–20% split train–test data and achieved a mean accuracy of 99.86% with 36 selected feature subsets by using RFF-CNN combination.

Santhakumar et al. [21] proposed a hybrid feature selection method combining two optimization algorithms, namely, ant colony optimization (ACO) and ant lion optimization (ALO). The proposed ant lion mutated ant colony optimizer feature selection technique achieved an accuracy of 95.45%, whereas ACO and ALO achieved 93.94%, and 90.91%, respectively. Sheikhpour et al. [22] implemented a feature selection method based on l_2,1_-norms and graph Laplacian as filter method to remove the redundant features. They applied SVM, Gaussian kernel density estimation-based classifier (GKDEC), KNN, and linear discriminant classifier (LDC) to classify the leukemia dataset. Among these four classifiers, the KGDEC and LDC obtained 100% accuracy with eight selected features

### 2.3. DLBCL

DLBCL [9] is the most common subtype of non-Hodgkin’s lymphoma (NHL), globally constituting up to 40% of all cases. In the United States alone, out of 100,000 people, it affects about 7 every year. It is fast-growing and is a very aggressive form of NHL. It is fatal if left untreated, but with appropriate treatment at the correct time, roughly about two-thirds of individuals can be cured.

Peng Zhou et al. [23] performed an online feature selection based on the dependency in KNN, called K-OFSD, and gained a maximum accuracy of 95.4% with 10 features using KNN as classifier for the DLBCL dataset. Chuanze et al. [24] presented a new feature selection technique, named rL-GenSVM, using relaxed lasso and generalized SVM (Gen-SVM). At first, they applied z-score normalization, then used relaxed lasso for the feature selection process, achieving 100% accuracy with eight selected features using Gen-SVM as classification algorithm. Yan et al. [25] proposed a tournament selection (TS) method and simulated annealing (SA) algorithm in combination with a coral reef optimization (CRO) to design a hybrid algorithm named BCROSAT. This BCROSAT algorithm gained a highest accuracy of 77.49% using KNN classifier as evaluator for the DLBCL dataset.

Bir-Jmel et al. [26] implemented a novel two-stage hybrid method named MWIS-ACO-LS. In the first stage, they used a combined approach of a new graph-based technique (named MWIS) and Fisher score, which acted as a filter method. In the second stage, a modified ACO and a local search (LS) algorithm using the neural network classifier were coupled together to assure the quality of the feature subsets. This method achieved the highest accuracy of 100% with six selected features for the DLBCL dataset. Authors of the work [14] implemented the proposed hybrid improved dragonfly algorithm (HIDA) method on DLBCL dataset and obtained 100% accuracy with an average count of 16 features.

Alirezanejad et al. [27] developed two filter methods, namely, XV, mutual congestion (MC), and two hybrid approaches combining these two methods, namely, XV-MC and MC-XV. At first, fitness for all the features was calculated with the proposed method and then they were given ranks. Next from the ranked list, on the basis of forward feature selection, 10 subsets of features were selected using Monte Carlo cross validation. Then, majority voting was applied over the best 10 features, and the highest accuracies achieved were 88%, 89%, 86%, and 85%, respectively, with NB used as classifier for the DLBCL dataset.

### 2.4. Prostate Cancer

Prostate cancer [10] is the second most frequently occurring malignancy in men worldwide, numbered at 1,276,106 fresh cases and being the cause of 358,989 deaths (which is around 3.8% of all cancer-based deaths in men) in 2018.

Liu et al. [28] implemented a hybrid GA with wrapper-embedded approach, namely, HGAWE, which combines GA and embedded regularization methods together. This feature selection method achieved a maximum accuracy of 94.17% with 22 attributes.

The authors of the work [26] implemented the proposed method MWIS-ACO-LS for the prostate cancer dataset, achieving a highest classification accuracy of 100% with 21 selected features. The work performed by Sun et al. [18] implemented the novel PDJE-AR method to the prostate cancer dataset, achieving an average accuracy of 91.2% with four features using C4.5 as classification algorithm.

Prabhakar et al. [29] proposed a transformation-based three-stage feature selection and classification of the prostate cancer dataset using wavelets. In the first stage, the wavelets are applied to mark important features. In the second stage, the four filter techniques—RFF, information gain, signal to noise ratio (SNR), and Fisher’s score—were applied to select the most relevant features. Finally, the four optimization techniques—migrating birds optimization algorithm (MBOA), marriage in honey bee optimization algorithm (MHBOA), salp swarm optimization algorithm (SSOA), and WOA—were applied. They achieved a best highest classification of 99.48% with 100 features with SNR + WOA method and used artificial neural network (ANN) as a classifier.

Cahyaningrum et al. [30] implemented another novel hybrid approach using ANN and GA. They used the principal component analysis method in the first stage for dimensionality reduction and selected 10 features with highest eigenvalues. Next, they used ANN along with GA, where GA was responsible for weights and bias optimization and ANN only computed feed-forward calculation. They achieved an accuracy of 76.47% by this method for the prostate cancer dataset. Deng et al. [31] presented a two-phase hybrid approach. In the first phase, they used the XGBoost algorithm, and in the second phase, they used a multi-objective optimization GA (MOGA) to obtain the relevant features, naming this method as XGBoost-MOGA. They ranked the features and taken relevant ones by using XGBoost algorithm and then used MOGA to mark the most important features among the selected ones from the first phase. They achieved 98% accuracy with 54 selected features using SVM as classifier for the prostate cancer dataset

### 2.5. Motivation

In a real-life scenario, datasets usually consist of a large number of features, and it becomes very difficult to differentiate noisy and useful features, as well as to interpret most contributing features. Due to the presence of redundant and unwanted features in the dataset, at times, classification of the entire dataset can lead to wrong decisions that can cause fatal damage in many cases, especially in the medical fields. Apart from this, the classification of a huge dataset without any feature selection is also computationally immensely costly. To outplay this issue, researchers have been working on different kinds of feature selection methods in order to obtain the optimal combination of features. Besides feature selection of medical datasets [32,33,34], researchers have utilized feature selection techniques in various domains such as handwritten script classification [35,36], facial emotion recognition [37], speech emotion recognition [38], and spoken language identification from audio signals [39,40] and have achieved notable classification accuracy improvement over the years. However, the two-phase filtering with the combination of four kinds of filter methods and classification, as well as one more phase of wrapper algorithm for the mentioned datasets have not been explored thus far. This motivates us to implement a tri-stage wrapper-filter feature selection framework for the disease datasets. To the best of our knowledge, this method is proposed for the first time for medical report-based disease classification.

## 3. Materials and Methods

**Figure 1:**
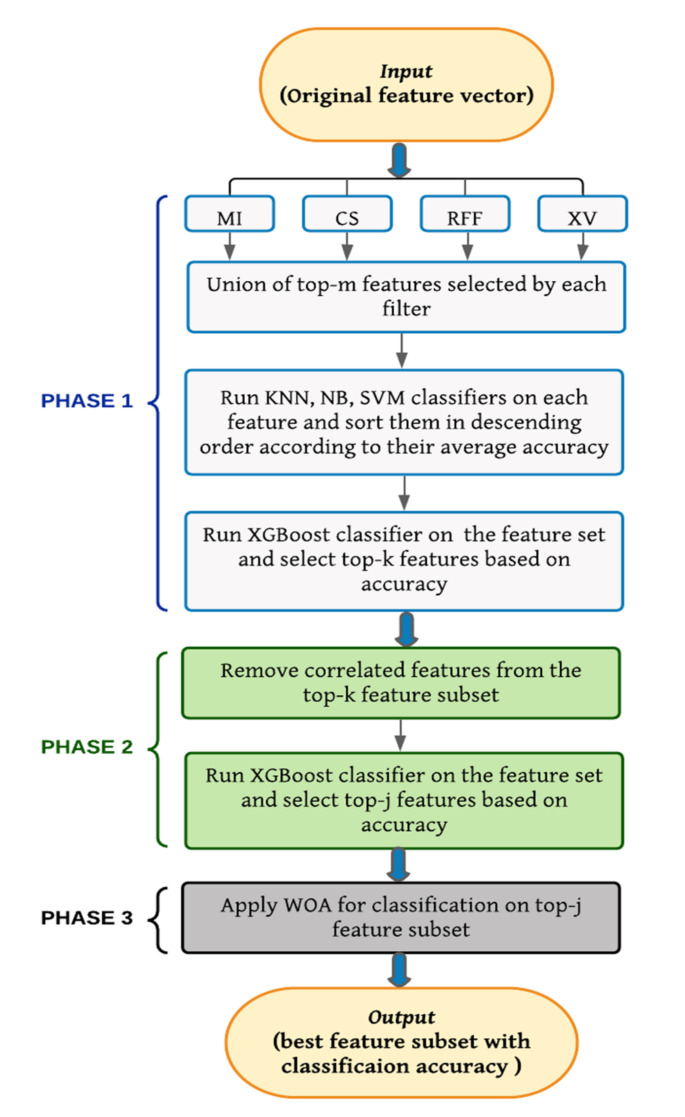
Flowchart of our proposed tri-phase hybrid wrapper-filter feature selection method.

In this section, the proposed tri-stage wrapper-filter feature selection method is described. The features are selected by a three-phase process. A flowchart of the proposed method is shown in Figure 1.

### 3.1. Phase 1

Different kinds of filter-based rankers were applied on the whole dataset, and sets of ranked lists were produced. The following four rankers produced the best results in terms of accuracy—MI, CS, RFF, and XV. A union of top-m features from each ranked set were taken. The value of m varied according to the different dataset sizes. A KNN classification algorithm was applied for preliminary assessment on the individual features from the union set, and the accuracy obtained by each feature was noted. Next, the other two classification algorithms, namely, SVM and NB, were applied on every feature from the union set, and their corresponding accuracies were noted.

When all the accuracies from the three classification algorithms were obtained, an average accuracy was taken for each feature and the features were sorted according to the descending value of the average accuracy. After this, the top-k features with the highest accuracy were selected for Phase 2. The value of k was determined by running the XGBoost classifier in the sorted union set, and it varied from 1 to the total number of features available in the sorted union set. The XGBoost classification algorithm was selected to determine the value of k as being a decision tree-based ensemble method, making the proposed method generalized for all the datasets. The value of k for which accuracy was not increasing (or was the same or the had decreased) after a certain value meant that many k number of features were chosen as the final feature subset. In this step, a large number of features were rejected, and only pertinent ones were selected. The ranker methods as well as classification algorithms used are described in detail in the following subsections.

#### 3.1.1. Ranker Methods Used

- MI:

In information theory and probability theory, the MI [41] of two particular variables is a measure of how mutually dependent the two variables are. It indicates the measurement of information that can be secured from a random variable by simply observing how it is changing with respect to another random variable. It is intently linked to the idea of entropy, as the depletion of uncertainty of a random variable can be calculated if another variable is studied. Hence, a high MI value specifies a large depletion of uncertainty, whereas a low value specifies a small depletion. If the MI is zero, it specifies that the two random variables are independent of each other. Let us say M and N are two random variables, then the MI between these two variables can be defined as

Ī(M; N) = Ɛ(M) − Ɛ(M | N)(1)

where Ī(M; N) is the MI for M and N, Ɛ(M) is defined as the entropy for M and N, and Ɛ(M | N) is defined as the conditional entropy for M given N.

- CS

The CS (χ^2^) [42] statistics measure the independence between a feature and its class. Let us say X is the number of examples, $X_{mn}$ is the number of samples of $C_{m}$ class within the nth interval, and $E_{mn}$ is the number of samples in the n^th^ interval. Now, the expected frequency of $X_{mn}$ is given by

(2) $$\chi^{2}=\overset{c}{\underset{m=1}{\sum }}\overset{K}{\underset{n=1}{\sum }}\frac{{\left(X_{mn}-E_{mn}\right)}^{2}}{E_{mn}}$$

where K is the number of the intervals and c is the number of classes. If pm is the probability of occurrence of event K, then the expected value Em = z ∗ pm, where z is the total number of events. Lower the value of CS, and more dependence is present between the features.

- RFF

Relief [43] was originally developed by Kira and Rendel. RFF is an extension of the relief algorithm developed after working on its limitations. Basic relief algorithm can classify only binary class problems, whereas RFF can classify multiclass problems. Relief algorithm essentially estimates the rank of features on the basis of to what extent the feature can differentiate between instances that are close to each other. RFF erratically selects an instance Ii, then it looks for k nearest hits of the same class hc and then for k nearest misses mc for each of the other classes. Then, RFF updates the weight of each feature by averaging the contribution of both all hits and misses. The primary difference between relief and RFF lies in choosing k hits and misses, which ensures the algorithm concerning noise to is more robust.

- XV

XV [27] calculates the distance between samples of one label from samples of another label within each feature. Higher distance or XV value means the feature is better and will be ranked higher. For a feature X, suppose there are n number of samples present of class i, which is indicated by $X_{i}$, and m number of samples present of class k, which is indicated by $X_{k}$. To determine the XV of feature X with respect to class, variance of n and m number of samples belonging to $X_{i}\;$and $X_{k}$ sample set needs to be calculated first, and the sum would be considered as the XV for feature X.

#### 3.1.2. Classification Algorithms Used

- KNN

KNN [44] is a supervised ML algorithm used to solve both classification and regression problems. KNN algorithm saves all the available data points, and when a new data point arrives, it classifies the new data point depending on the similarity measures (e.g., distance function). Majority voting is used to classify the new data point, and it is assigned the class in which most of its neighbors are located. Accuracy achieved by KNN varies with the number of neighbors or the value of k used by the algorithm.

- SVM

In 1963, Vladimir Vapnik and Alexey Chervonenkis developed the original SVM algorithm, which can classify only linearly separable data, and in the 1990s, Vapnik used kernel trick to extend the usage of this classification method, which can separate non-linearly separable classes [45]. SVM is a supervised ML method mostly used in classification problems. Let us say there is n number of features present of two classes; then, each feature is treated as a data point, and using the feature value as co-ordinate value, they are plotted in n-dimensional space. Then, a hyper-plane is drawn to put a margin between the two classes so that features belonging to each class reside on the opposite side of the hyper-plane. In this way, the classification is achieved by detecting the hyper-planes, which help to distinguish the two classes accurately.

- NB

NB [46] is a supervised ML algorithm mostly used for classification problems. In statistics, NBs are “probabilistic classifiers” built by applying Bayes’ theorem. NB classifier has a presumption about the features that they are independent of each other, which means presence of any particular feature does not have impact on any other feature of the class. This is one of the simplest forms, and to achieve higher accuracy, they are coupled with kernel density estimation.

- XGBoost

XGBoost [47] or extreme gradient boosting is a popular and commonly used ML algorithm that is based on gradient boosting technique and an extension to gradient boosted decision trees (GBM). It was developed by Tianqi Chen, and it helps in enhancing performance and speed in tree-based (sequential decision trees) ML algorithms. It is a faster algorithm as it has parallel and distributed computing methods. It has some inbuilt qualities that make it different from others, such as cache optimization, variety of regularizations to reduce overfitting, auto tree pruning, and built in cross-validation.

### 3.2. Phase 2

The k-features obtained from the Phase 1 are input to this phase. In this phase, correlation is measured among the features.

Pearson correlation coefficient (PCC) was used to measure the pairwise correlation of all 50 features. PCC can be described as a measurement of strength of a linear association between two features or variables. It is generally notified by r, where r=1 means the features have a perfect positive correlation, r=−1 means they have a perfect negative correlation, and r=0 means there is no correlation present between the attributes. Let us say there is i number of instances present for two variables a and b; then, PCC between a and b can be calculated as

(3) $$r=\frac{\sum \left(a_{i}-\alpha \right)\left(b_{i}-\beta \right)}{\sqrt{\sum {\left(a_{i}-\alpha \right)}^{2}\sum {\left(b_{i}-\beta \right)}^{2}}}$$

where $a_{i}$ and $b_{i}$ are any two random instances; α and β are the mean values for the features a and b, respectively; and r is the PCC value between a and b.

A correlation matrix is calculated among all features and with respect to class as well. If two features are highly correlated, then the one that is having greater r value with respect to the class is kept and the second one is rejected. Then, the XGBoost classifier (as explained in Section 3.1.2) is applied on the non-correlated feature set, and the same logic of Phase 1 is applied to select top j features. These j numbers of features are then sent to Phase 3. This process helps to reduce the less informative features.

### 3.3. Phase 3

The top j features obtained from Phase 2 are passed to Phase 3, and a different number of search agents, ranging from 50 to 70, are deployed for different datasets, and maximum number of iterations is set to 100. This algorithm is run for 20 times, and average accuracy and feature number are noted down as final accuracy.

#### 3.3.1. Whale Optimization Algorithm

WOA is a nature-inspired meta-heuristic algorithm proposed by Majdi [48]. Researchers are using it as a wrapper-based feature selection method in various domains [29,49,50]. It follows the bubble-net feeding in the foraging behavior of the humpback whales. They swim in a ‘6′-shaped path and hunt close to the surface [51]. They trap the prey in a net of bubbles. The algorithm mimics the behavior of the humpback whales in two main phases—exploitation and exploration phases. In the exploitation phase, the prey is encircled in spiral bubble-net attacking mode, and in the exploration phase, there is a search for random prey. Each phase is described in detail in the following subsections.

#### 3.3.2. Exploitation Phase

At first, the location of the prey is recognized by the humpback whales, and they then encircle the prey. WOA always presumes that target prey is the best candidate solution or at least it the closest to the optimal, as the optimal design position in the search space is not known in advance. After the best search agent is selected, other search agents will also therefore try to update their location according to the location of the best search agent. For a location to be updated, Equations (4) and (5) are used.

(4) $$\vec{B}=\left|\vec{J}.\vec{X^{*}}\left(p\right)-\vec{X}\left(p\right)\right|$$

(5) $$\vec{X}\left(p+1\right)=\vec{X^{*}}\left(p\right)-\vec{K}.\vec{B}$$

where p, $X^{*}$, and X represent the current iteration; best solution’s position vector calculated thus far; and the current solution X, which is the position vector, respectively. J and K are coefficient vectors, and the rest are normal mathematical interpretations such as | |, which is the absolute value and ·, which is an element-by-element multiplication. The value of $X^{*}$ would be updated in each iteration if any better solution is present. The coefficient vectors, J and K, are calculated as in Equations (6) and (7).

(6) $$\vec{K}=2\vec{s}.\vec{v}-\vec{s}$$

(7) $$\vec{J}=2.\vec{v}$$

where, v is a random vector in $\left[0,1\right]$, and s decreases linearly from 2 to 0. The position of the solution that is closest to the best solution is controlled by adjusting the values of the vectors K and J.

Next, the shrinking encircling behavior is simulated by decreasing the value of s in Equation (6) according to Equation (7).

(8) $$s=2-p\frac{2}{MaxIter}$$

where, p and MaxIter represent the iteration number and the maximum number of allowed iterations, respectively. After the shrinking is performed according to the value of s, the distance between the solution (X) and the leading solution ($X^{*}$) is next calculated to achieve the spiral-shaped path. Equation (9) provides the spiral equation created between the current solution and the best (leading) solution.

(9) $$\vec{X}\left(p+1\right)=B^{\prime }.e^{bl}.\cos\left(2\pi l\right)+\vec{X^{*}}\left(p\right)\;$$

where, b defines the spiral’s shape, l is a random number in $\left[-1,\;1\right]$, and B is the distance between a whale X and a prey

$$\left(\vec{B}=\left|\vec{X^{*}}\left(p\right)-\vec{X}\left(p\right)\right|\right).$$

To model the shrinking encircling mechanism and the spiral-shaped path, we assumed a probability of 50% to choose between them during the optimization, as shown in Equation (10).

(10) $$\vec{X}\left(p+1\right)=\left\{\begin{array}{l} Shrinking\;Encircling,\;\;\vec{X}\left(p+1\right)=\vec{X^{*}}\left(p\right)-\vec{K}.\vec{B}\;\;\;if\;t<0.5\\ Spiral\;Shaped\;Path,\;\;\vec{X}\left(p+1\right)=B^{\prime }.e^{bl}.\cos\left(2\pi l\right)+\vec{X^{*}}\left(p\right)\;\;\;if\;t\geq 0.5 \end{array}\right.$$

where, t represents the probability and is a random number in $\left[0,\;1\right]$.

#### 3.3.3. Exploration Phase

To enhance the performance of the exploration phase in WOA, instead of updating the solutions on the basis of the position of the best solution obtained thus far, we utilized a randomly chosen solution to update the solutions positions. Thus, any random value is used for vector K, where K is greater than 1 or less than −1. This forces a solution to be distant from the best-known search agent. Equations (11) and (12) model this mechanism mathematically.

(11) $$\vec{B}=\left|\vec{J}.\vec{X_{r}}-\vec{X}\right|$$

(12) $$\vec{X}\left(p+1\right)=\vec{X_{r}}-\vec{K}.\vec{B}$$

where, $\vec{X_{r}}$ is a randomly chosen whale.

Let us say there is a total of ‘n’ number of features present in the dataset, and they are divided into feature subsets. This individual feature subset is considered as a position of a whale. The goal is to choose less number features in the solution subset and achieve higher classification accuracy using KNN as a classifier. A fitness function is used to evaluate each solution subset. This fitness function depends on two objectives: number of features in the solution subset and the solution’s accuracy. Algorithm 1 describes the algorithm of WOA.
**Algorithm 1** Algorithm of WOA*Input: Number of whales (n), Max_Iter* *Output: Prey or the fittest whale* Generate Initial Population Xi (i=1, 2, …, n) Calculate the objective value of each solution     $X^{*}=the\;best\;solution$ **while** (p<Max_Iter) **for** each solution Update s, K, J, l, and t **if** (t<0.5) **if**$\;(\left|K\right|<1$) Update the current solution’s position by Equation (5) **else if**$(\left|K\right|>1$) Select a random solution (Xr) Use Equation (12) **end if** **else if** (t>=0.5) Update the current solution’s position by the Equation (9) **end if** **end for** Check whether there is any solution present beyond the search space and update it Calculate each solution’s fitness $\mathrm{If}\;a\;\mathrm{better}\;\mathrm{solution}\;\mathrm{is}\;\mathrm{found},\;\mathrm{update}\;X^{*}$     p=p+1 **end**
***while*** return X*

### 3.4. Dataset Details

The proposed tri-phase hybrid wrapper-filter feature selection model has been used to experiment on the arrhythmia [52], leukemia [53], DLBCL [54], and prostate cancer [55] datasets taken from the UCI ML Repository and Biolab Repository. The detailed information regarding the datasets used in the present work is mentioned in Table 1. The proposed tri-phase hybrid wrapper-filter feature selection method has been implemented using Jupyter Notebook and Google Colaboratory. RFF has been implemented using scikit-rebate [56], and WOA has been implemented using a PY-FS package [57].

Table 1 summarizes the number of attributes, instances, and class distribution of the datasets used in this work. Arrhythmia dataset, taken from UCI repository, has 16 different classes ranging from 1 to 16, among which 2–15 are affected classes and 1 is non-affected. Class 16 is unrecognized, but in all the related work mentioned in this study, the authors have considered class 16 as affected, which is why in this work class 16 is also considered as affected class. Thus, the dataset has been reclassified into two classes, non-affected (class 1) consisting of 245 samples and affected (2–16) consisting of 207 samples, and it has 279 features. Leukemia dataset is taken from Biolab Repository, and it has 5147 features that are lesser than the original dataset [58], as features that are not present in at least one sample have been removed. It has 72 instances, among which 47 are of ALL and 25 are of AML class. The DLBCL dataset is also taken from the Biolab Repository. It has samples of two classes, one being DLBCL class and another being follicular lymphoma (FL) class. Although DLBCL and FL both are B-cell lineage malignancies, their clinical approach and treatment method are totally different. Prostate cancer dataset, taken from Biolab Repository, consists of 52 samples of tumor tissue and 50 samples of normal tissue, having a total of 12,532 features.

## 4. Results and Discussion

This section is divided into subsections. The first subsection contains information about all parameters tuning used to implement the proposed method. In the second subsection, a detailed analysis of the results obtained in each phase of the proposed method is described. In the third subsection, comparative study was performed with the state-of-the-art methods mentioned in the literature survey section in terms of the number of features used and accuracies obtained. In the fourth subsection, a statistical significance test result with respect to accuracy between state-of-the-art methods and proposed method is described. Finally the fifth subsection, a comparative study was performed again with three other non-medical UCI benchmark datasets in terms of the number of features used and accuracies obtained.

### 4.1. Parameter Tuning

In Phase 1, the only parameter value that has been varied is the value of m. For the arrhythmia dataset, the value of m was varied from 50 to 70 with an interval of 5, i.e., 50, 55, 60, 65, 70, and 75, and six feature subsets were obtained. Then, the four classifiers were applied on the six subsets, and after taking mean accuracy, we sorted the features according to their achieved accuracy. The XGBoost classification algorithm was applied on the six sorted feature subsets. It was observed that for all six feature subsets, after the first 60 features, the accuracy either decreased or remained the same. Thus, the value of m was selected as 50 for the arrhythmia dataset in Phase 1. In the same way, m was varied from 100 to 150 for the leukemia dataset, 150 to 200 for the DLBCL dataset, and 200 to 250 for the prostate cancer dataset, keeping an interval value of 10 for all three datasets. The four classifiers were used with default parameter values, and in Phase 2, similarly, the XGBoost classification algorithm was applied with default parameter values.

In Phase 3, there were features remaining in the range of [20,25] for all the four medical datasets. Thus, the WOA was applied for the arrhythmia dataset first. Number of search agents was varied from 30 to 100 with an interval of 5, whereas the number of iterations was fixed at 100. It was observed that the best optimal result was obtained when the number of search agents was 70 and the optimal solution converged by 100 iterations. In the same way, for DLBCL, leukemia, and prostate cancer datasets, the number of optimal search agents was found to be 50, 60, and 50, respectively, with a fixed iteration number of 100. The value of K in KNN varied from 3 to 7 for the datasets, and K = 5 gave better results for both arrhythmia and prostate cancer datasets. For the DLBCL and leukemia datasets, K = 4 produced better results. After obtaining the ideal values for the number of agents, the number of iterations, and K value in KNN, we applied the WOA 20 times on the four datasets, and the average value was taken in terms of both accuracy and features as the number of features varied in the range of [1,10] for each run. Table 2 represents the summary of parameter details used in each phase.

### 4.2. Experimental Outcomes and Analysis

Before evaluation, some preprocessing was applied to the aforementioned datasets. The arrhythmia dataset had multiple missing values present for all features that were replaced by mean values respective of classes. Other than this, as a part of preprocessing, all four disease datasets were standardized and then normalized.

After the preprocessing, three ML algorithms—KNN, SVM, and NB—were applied on the datasets with 10-fold cross validation to compare the accuracy with and without any feature selection. At Phase 1, four filter methods—MI, CS, RFF, and XV—were applied separately to rank the features. Then, a union of top m features were taken, a KNN classification algorithm was applied on the individual feature obtained by the union, and their corresponding accuracies were noted. The NB and SVM classifiers were applied, and the accuracy for individual features was noted. Next, the mean accuracy of features were calculated and sorted according to the descending value of accuracy. It is to be noted that the value of m was different for each medical dataset. Afterward, the top k features were selected by running XGBoost algorithm on the union set. These top k features were passed to Phase 2 for further processing, and in this Phase 2, the feature subset was reduced again depending on how much correlated the features were. If any two features were highly correlated (PCC value>0.7), the second feature was rejected and the first one was kept. Then, the XGBoost classification algorithm was applied on the selected non-correlated feature set and top j features were selected. To check the best value of k and j, we applied XGBoost classification algorithm along with 10 cross-validation (CV) in each phase. For Phase 1, the XGBoost algorithm was applied on the union set, taking values from 5 to the total feature number of the union set (say, s), with intervals of 5, say, 5, 10, 15, …, s to find after which value of k the accuracy obtained was either decreasing or remaining the same. In the same way, the value of j for Phase 2 was also determined by varying the value of j from 1 to the total number of selected features after correlated features were discarded. In Table 3, detailed values of m, k, j, and correlated features and non-correlated features are mentioned. The reduced feature set of j features was considered to be passed on to the wrapper method. In Phase 3, the top j features were selected from the feature set obtained by Phase 2, and WOA was applied on the selected feature set.

Table 3 summarizes all the features used and obtained in Phase 1 and Phase 2. In Phase 1, a union of 50, 100, 150, and 200 features were taken by each filter method (namely, CS, MI, RFF, and XV) participating in the union for arrhythmia, leukemia, DLBCL, and prostate cancer datasets, respectively. Then, as mentioned previously, after applying three classification algorithms, we sorted the features according to the non-increasing mean value of accuracies. In order to find the optimal subset, we applied the XGBoost algorithm on the union set. Then, for all the datasets, if their corresponding accuracies were varying in a sine wave manner up to a certain number of feature subsets, then the accuracy would either remain the same when more features are added in the feature subset or accuracy would decrease. Thus, the number of features for which the accuracy was in a non-increasing state were found to be 60, 70, 50, and 75, respectively, for all the four datasets, and these features are then sent to Phase 2.

In Phase 2, the correlation value was calculated and the highly correlated features were discarded. In this phase, 25 out of 60 features obtained by Phase 1 were marked as highly correlated and discarded for the arrhythmia dataset. The XGBoost algorithm was again applied on the remaining 35 features, and it was observed that the accuracies were varied for the first 20 features, after which they were decreasing. Those 20 features were selected and sent to Phase 3. In the same way, the first 23, 23, and 25 top features were obtained by Phase 2 for leukemia, DLBCL, and prostate cancer datasets, respectively. Finally, in the third phase, the WOA was applied on the top j features obtained from Phase 2. Table 4 shows the comparison between accuracies obtained over the whole datasets by applying three classification algorithms (KNN, SVM, and NB) and the number of best features and accuracies obtained after applying the proposed tri-stage method.

**Figure 2:**
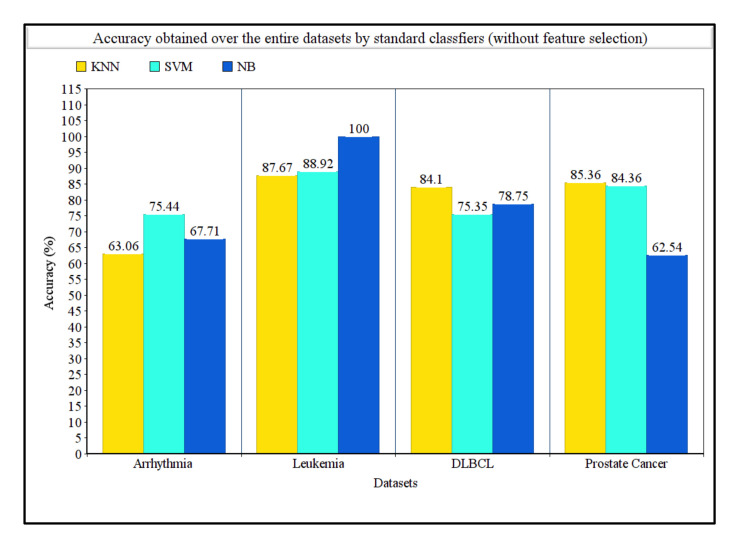
Comparison of accuracies obtained on four disease datasets using KNN, SVM, and NB classifiers without any feature selection.

**Figure 3:**
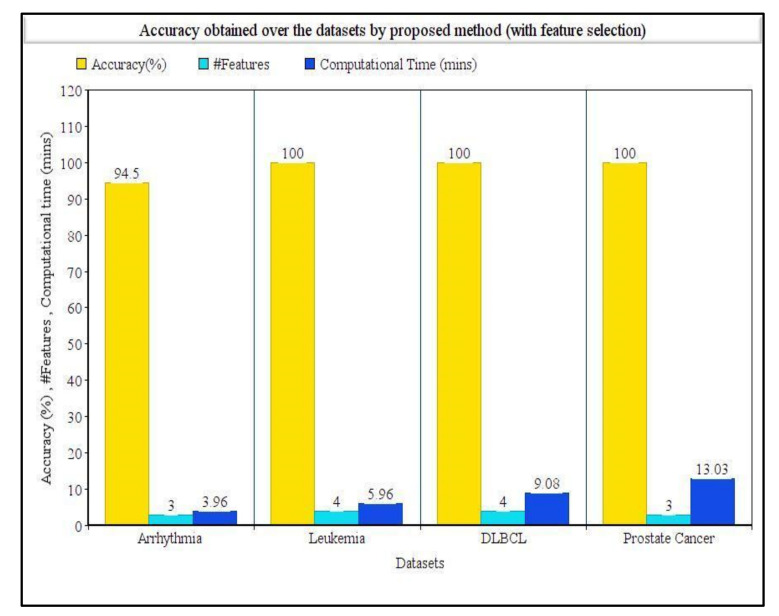
Comparison of accuracies, number of features, and computational time obtained on four disease datasets using our proposed tri-stage wrapper-filter feature selection method.

Table 4 shows the detailed results obtained over the four datasets taken into consideration. It clearly shows the proposed method succeeded in determining the best features along with best accuracies. For the arrhythmia dataset, the SVM classifier achieved the highest accuracy over the whole dataset, whereas the proposed method achieved 94.50% accuracy with only three features. For the leukemia dataset, the NB classifier achieved 100% accuracy, but with the proposed method, 100% accuracy was also obtained with only four features, which was remarkably helpful. Similarly, for both DLBCL and prostate cancer datasets, the KNN classifier achieved the highest accuracies of 84.10% and 85.36%, respectively, without using any feature selection procedure. On the other hand, our proposed method achieved a 100% accuracy for both the datasets utilizing only four and three features, respectively. Table 4 summarizes the comparison in terms of computation time taken by our proposed method with and without using the feature selection method. For arrhythmia, leukemia, DLBCL, and prostate cancer datasets, the computation times needed by our proposed method were 238, 358, 545, and 782 s, respectively. The computation times needed to run the original feature set (i.e., without using the feature selection method) were found to be 42.8, 43.9, 47.5, and 55.6 s for arrhythmia, leukemia, DLBCL, and prostate cancer datasets, respectively. Figure 2 represents the classification accuracies obtained by KNN, SVM, and NB classifiers on the whole dataset without any feature selection, and Figure 3 represents the classification accuracies obtained by using our proposed feature selection method along with the number of features selected for each dataset.

In order to calculate the highest accuracies and optimal feature sets obtained by each phase, we applied the XGBoost algorithm on the feature set achieved by Phase 1 and Phase 2 while determining the value of k and j for Phase 1 and Phase 2. As mentioned previously, both the values of k and j varied from 1 to the number of features present in the selected subset to find the value for which the accuracy obtained was the highest. Then, say the accuracy was highest for n = 15, the XGBoost algorithm would then again be applied for n ± 5 (i.e., from 10 to 20) to find the exact value of n where the accuracy was maximum. Table 5 shows the accuracy, precision, recall, and F1_Score of the selected features of Phase 1 and Phase 2 of arrhythmia, DLBCL, leukemia, and prostate cancer datasets.

**Figure 4:**
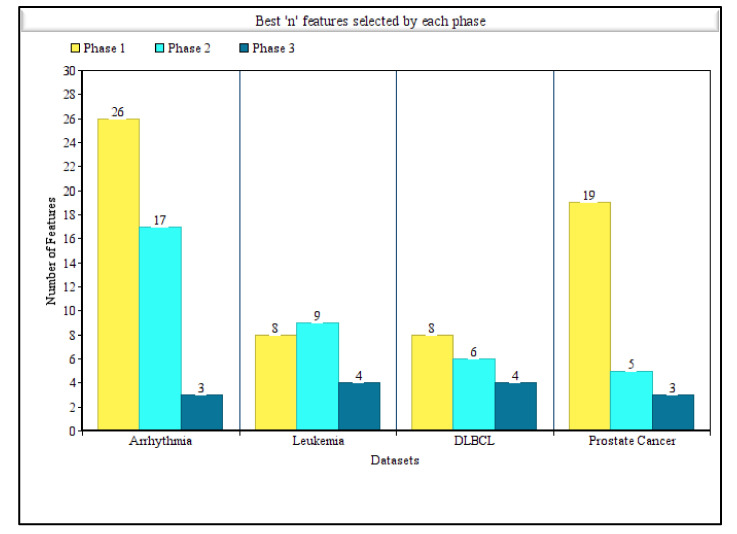
Comparison of number of features obtained by each phase of our proposed tri-stage wrapper-filter feature selection method for all the four disease datasets considering the highest accuracy achieved in each phase.

Table 5 also summarizes the best n features selected by Phase 1 and Phase 2 separately. It is noticeable that except for the DLBCL dataset, for all other datasets, the number of optimal n features decreased exceptionally, even though the measures were not almost the same. This accentuates the idea of Phase 2 that the correlated features were unnecessary and that this model can achieve either same or better accuracy using any other measure when the redundant features are discarded. For the arrhythmia dataset, after the execution of Phase 1, a subset of 26 features attained an accuracy of 96.46%, and after the execution of Phase 2, a subset of 17 features attained comparatively lesser accuracy, whereas precision and other measures were almost same. For the leukemia dataset, eight features were selected by Phase 1 and six were selected by Phase 2 as the best n features. Although accuracy increased by 1% after Phase 2, the precision, recall, and F1_measure decreased. For the DLBCL dataset, the best n feature numbers were eight and nine, respectively, for Phase 1 and Phase 2. For the DLBCL dataset, all the measures achieved by Phase 2 were greater than Phase 1. Finally, for the prostate cancer dataset, the difference between the number of features, for which the highest accuracy was achieved by Phase 1 and Phase 2, was huge. However, almost the same accuracy, precision, recall, and F1-Score values were achieved while using nearly four times less features. Figure 4 represents the comparison of best n features obtained by each phase when highest accuracies were achieved for all the four datasets.

### 4.3. Comparison with State-of-the-Art Methods

Table 6, Table 7, Table 8 and Table 9 show the comparison (in terms of both number of features and accuracies obtained) between some past methods and the proposed tri-stage wrapper-filter feature selection method for arrhythmia, leukemia, DLBCL and prostate cancer datasets, respectively.

For the arrhythmia dataset, Sahebi et al. [13] obtained the highest accuracy of 99.02%, whereas the proposed method achieved 94.50%. However, the mentioned work also chose a subset of 135 features, whereas the proposed method selected only three features, being is 45 times less than the former. Among the other mentioned works, Wang et al. [16] achieved an accuracy of 98.68% with a subset feature of 89, which was again almost 30 times larger than the number of features chosen by our proposed method. It can be seen from Table 6 that all the other works achieved less accuracy while utilizing more features than our proposed method. The authors of [11,14,15] selected a very large number of features (236, 135, and 169 features, respectively) as an optimal feature set and achieved 82.96%, 74.77%, and 88.72% accuracies, respectively. Authors of the work [12] succeeded in selecting a comparatively lesser number of features (30 features), but the accuracy obtained was 85.58%.

For the leukemia dataset, Sheikhpour et al. [22] obtained the highest accuracy of 100%, and the proposed method also achieved the same. However, the number of features selected by the proposed method was comparatively less. The authors selected eight features, and the proposed method selected only four features. Kilicarslan et al. [20] achieved a notable accuracy of 99.86% using 36 features. Santhakumar et al. [21] obtained 95.45% accuracy, but they did not mention any number of features in particular. Authors of works [17,19] achieved almost the same accuracy of 91.05% and 90.88%, respectively, while using the same number of optimal features (27 features). Sun et al. [18] succeeded in selecting only seven features and attained only 87.5% accuracy for the leukemia dataset.

For the DLBCL dataset, the work mentioned in [14,26,30] attained an accuracy of 100% but the number of features selected by the authors were greater than the number of features selected by our proposed method. They utilized 16, 6, and 8 features, respectively, whereas the proposed method selected only four features as the optimal feature set. All the other works mentioned gained lesser accuracy than the proposed method. The authors of both [23,32] selected 10 significant features to attain a maximum accuracy of 95.4% and 89%, respectively. Yan et al. [25] achieved 77.49% accuracy using a hybrid algorithm named BCROSAT.

For the prostate cancer dataset, Bir-Jmel et al. [26] achieved an accuracy of 100%, selecting 21 features. However, the proposed method also obtained the same accuracy while utilizing only three features. Although the work performed by the authors in [29,31] attained accuracies of 99.48% with 100 features and 98% with 98 features, respectively, they chose 33 times and 28 times more features than the proposed method, respectively. Sun et al. [18] attained an optimal feature set with only four features, but the accuracy also decreased. They attained 91.2% accuracy with the selected four features. The work conducted by the authors of [28,30] implemented different hybrid GAs and achieved accuracies of 94.17% with 22 features and 76.4% accuracy with 10 features, respectively.

### 4.4. Statistical Significance Test

Statistical significance test was also performed to prove that the results of the proposed method were statistically significant as compared to the other state-of-the-art algorithms mentioned in this paper. Statistical tests provide a means to make quantitative decisions about any process [59]. The goal was to ascertain whether there was sufficient evidence to ‘reject’ a conjecture or hypothesis about the process [60,61]. The conjecture is called the null hypothesis. For our case, the null hypothesis stated that the two sets of results had the same distribution. In order to reject the null hypothesis, we performed one-sample *t*-test [62] with two different significance levels, and the results are given in Table 10. Both *t*-values and *p*-values were calculated with respect to classification accuracy. From Table 10, it can be concluded that our proposed method is statistically significant in comparison to other methods for all the four datasets at the 0.10 level of significance.

### 4.5. Results on Other UCI Datasets

Other than medical datasets, the proposed method was applied on three UCI benchmark datasets—Ionosphere [63], Krvskp [64], and Sonar [65]—from different domains.  Table 11 summarizes the dataset details of three benchmark datasets. Table 12 shows the comparison (in terms of both number of features and accuracy obtained) between some past methods [1,2,33,34,59,66] and the proposed method for the said datasets.

The proposed method achieved an average accuracy of 95.77% with four features for the Ionosphere dataset, whereas the authors of paper [2,59] both obtained almost the same accuracy of 98.56% with seven features. The authors of [66] also achieved a higher accuracy of 97.18%, but they also used more features than our proposed method. The authors of [1] achieved lesser accuracy while utilizing more features. For the Krvskp dataset, all the previous works [2,33,34,59] achieved higher accuracy, but they consumed a very high number of features as well. The lowest number of features among them was 15, which was obtained by the authors of [59] with an accuracy of 97.81%, whereas the proposed method achieved an 95.15% accuracy with only four features. The authors of paper [2] obtained an accuracy of 99.06% with 32 features, which was eight times more than the number of features obtained by our proposed method. For the Sonar dataset, the authors of [34] obtained the highest accuracy of 100% with 16 features, whereas our proposed method achieved 92.85% accuracy with only four features. However, it was obvious that the accuracy achieved in [34] was almost perfect (being 100%), but it is to be noted that the reduction in the number of features (described in [34]) was found to be four times lesser by our proposed method. Among other methods, authors of the method reported in [66] obtained an accuracy of 97.62% with 51 features, and the method mentioned in [59] achieved almost the same accuracy as reported by our proposed method but they used 22 features as well. The best results achieved by our proposed method for the Ionosphere, Krvskp, and Sonar datasets were 98.05% accuracy with seven features, 98.75% accuracy with nine features, and 94.75% accuracy with eight features, respectively.

## 5. Conclusions

In this work, a new tri-stage feature selection was proposed to reduce the attribute set of the high-volume dataset by selecting a subset of the original features, which is essential. If any highly important feature is missed, it may result in wrong classification, which is especially undesirable, particularly in medical data. The proposed method successfully marked the important features and achieved 100% accuracy for all the three cancer datasets and 94.50% accuracy for the arrhythmia dataset. This method uses a combination of four filter methods (MI, CS, RFF, and XV) at the Phase 1 along with three classification algorithms (KNN, SVM, and NB) so that every possible feature with highest accuracy independent of both filter methods and classification algorithms is selected. Then, the XGBoost classification algorithm is applied on the union set, and k number of top features is selected on the basis of accuracy. At Phase 2, correlation is measured using PCC among the top k features obtained by Phase 1, and highly correlated features are discarded, ensuring that maximum relevance minimum redundancy policy is present among the feature subset. Again, XGBoost is applied, and top j features are obtained and sent to Phase 3. In the final phase (i.e., Phase 3), a wrapper method, WOA, is used to finalize the optimal feature subset. Arrhythmia, leukemia, DLBCL, and prostate cancer, four medical datasets of varying dimensionality, were used for experimentation. The least ranked features by the ensemble were the ones that had least importance in the dataset in terms of information, dependence, and distance. In each phase, feature set was reduced, and after whole implementation was performed, number of features in the optimal feature set was reduced almost 150 times for arrhythmia, 1286 times for leukemia, 1767 times for DLBCL, and 4177 times for prostate cancer datasets from the original feature set. Thus, the proposed method also provided the information about the noisy, redundant, or unnecessary features in each phase, which were then discarded. Moreover, the accuracies achieved for each dataset were found to be satisfactory.

However, the datasets considered in the present work have a very limited amount of samples. For all the cancer datasets, the attributes are very minuscule compared to the enormous attribute range [67]. More particulars of different ages, sexes, heredity information, etc. are needed to be included in all the datasets. Along with the dataset update, there is also a large amount of room for exploration of ensemble techniques. There are numerous possible ways of combining multiple filter-wrapper methods, and therefore the experimentation prospect is huge regarding the ensemble. Moreover, both leukemia and DLBCL datasets are imbalanced, which, in future, can be handled by generating reliable synthetic data using various over-sampling or under-sampling methods. Although all the four diseases used in this work are incurable, they can be controlled if diagnosed in time. Thus, the study is expected to help minimizing diagnosis time as well as to help the community economically. Other than limited data present in the datasets, one of the shortcomings of this algorithm is the computational complexity required to compute accuracy of all the datasets on the basis of each individual feature in Phase 1 and to perform the WOA algorithm. In the future, this method can be applied on other publicly available medical datasets and can be combined with other available wrapper-filter methods while using different classification algorithms.

## Figures and Tables

**Table 1:** Details of four medical datasets used in this work.

Sl. No.	Dataset	Total Number of Attributes	Total Number of Instances	Class Distribution
1.	Arrhythmia	279	452	Non-affected: 245 Affected: 207
2.	Leukemia	5147	72	ALL: 47 AML: 25
3.	DLBCL	7070	77	Non-DLBCL: 19 DLBCL: 58
4.	Prostate cancer	12,532	102	Normal tissue: 50 Prostate tumor: 52

**Table 2:** Parameters details of Phase 1 and Phase 3 for the four medical datasets.

Dataset	Parameter Details		
	Phase 1	Phase 3	
	Value of *‘m’*	#Search Agents	Value of ‘K’ in KNN
**Arrhythmia**	Range: 50–70 Interval value: 5 Final value: 50	Range: 30–100 Interval value: 5 Final value: 70	5
**Leukemia**	Range: 100–150 Interval value: 10 Final value: 100	Range: 30–100 Interval value: 5 Final value: 50	4
**DLBCL**	Range: 150–200 Interval value: 10 Final value: 150	Range: 30–100 Interval value: 5 Final value: 60	4
**Prostate cancer**	Range: 200–250 Interval value: 10 Final value: 250	Range: 30–100 Interval value: 5 Final value: 50	5

**Table 3:** Details of the features used to obtain the optimal feature subset in each phase.

Dataset	Phase 1		Phase 2		
	Number of Features Used for Union (m)	Number of Features Selected (k)	Number of Discarded Correlated Features	Number of Non-Correlated Features	Number of Features Selected (j)
Arrhythmia	50	60	25	35	20
Leukemia	100	70	46	24	23
DLBCL	150	50	26	24	23
Prostate cancer	200	75	50	25	25

**Table 4:** Comparison in terms of accuracy, number of features selected, and computation time while experimenting on four disease datasets with and without applying our proposed feature selection method.

Dataset	Accuracy (%) Obtained by the Proposed Method (with Feature Selection)				Accuracy (%) on the Entire Dataset (without Feature Selection)			
	Original #Features	#Features Selected	Accuracy (%)	Computation Time (s)	KNN	SVM	NB	Computation Time (s)
**Arrhythmia**	279	3	94.50	238	63.06	75.44	67.71	42.8
**Leukemia**	5147	4	100	358	87.67	88.92	100	43.9
**DLBCL**	7070	4	100	545	84.10	75.35	78.75	47.5
**Prostate cancer**	12,532	3	100	782	85.36	84.36	62.54	55.6

**Table 5:** Comparison of accuracy, precision, recall, and F1_Score obtained over the disease datasets with XGBoost classifier and number of best n features obtained by Phase 1 and Phase 2.

Dataset	XGBoost Classifier					
		Accuracy (%)	Precision (%)	Recall (%)	F1_Score (%)	No. of Best n Features
Arrhythmia	*Phase 1*	96.46	94.21	98.57	96.34	26
	*Phase 2*	96.24	93.82	98.41	96.06	17
Leukemia	*Phase 1*	96.25	90	87.5	88.73	8
	*Phase 2*	97.32	80	80	80	6
DLBCL	*Phase 1*	92.14	68.33	70	69.15	8
	*Phase 2*	93.39	73.33	70	71.62	9
Prostate cancer	*Phase 1*	95.18	96.57	94.89	95.72	19
	*Phase 2*	94.18	96.57	93.55	95.03	5

**Table 6:** Comparison in terms of accuracies (%) and number of selected features obtained by the proposed method with some state-of-the-art methods for the arrhythmia dataset (highest accuracy and lowest #features are highlighted in bold).

Dataset	Method	No. of Features Selected	Classification Accuracy (%)
**Arrhythmia**	Xu et al. [11]	236	82.96
	Singh et al. [12]	30	85.58
	Sahebi et al. [13]	135	**99.02**
	Cui et al. [14]	169	74.77
	Kadam et al. [15]	92	88.72
	Wang et al. [16]	89	98.68
	**Proposed**	**3**	94.50

**Table 7:** Comparison in terms of accuracies (%) and number of selected features obtained by the proposed method with some state-of-the-art methods for the leukemia dataset (highest accuracy and lowest #features are highlighted in bold).

Dataset	Method	No. of Feature Selected	Classification Accuracy (%)
**Leukemia**	Wang et al. [17]	27	91.05
	Sun et al. [18]	7.6	87.5
	Khamess et al. [19]	27.91	90.88
	Kilicarslan et al. [20]	36	99.86
	Santhakumar et al. [21]	NA	95.45
	Sheikhpour et al. [22]	8	**100**
	**Proposed**	**4**	**100**

**Table 8:** Comparison in terms of accuracies (%) and number of selected features obtained by the proposed method with some state-of-the-art methods for the DLBCL dataset (highest accuracy and lowest no. of features are highlighted in bold).

Dataset	Method	No. of Features Selected	Classification Accuracy (%)
**DLBCL**	Peng Zhou et al. [23]	10	95.4
	Chuanze et al. [30]	8	**100**
	Yan et al. [25]	NA	77.49
	Bir-Jmel et al. [26]	6	**100**
	Cui et al. [14]	16	**100**
	Alirezanejad et al. [32]	10	89
	**Proposed**	**4**	**100**

**Table 9:** Comparison in terms of accuracies (%) and number of selected features obtained by proposed method with some state-of-the-art methods for the prostate cancer dataset (highest accuracy and lowest no. of features are highlighted in bold).

Dataset	Method	No. of Features Selected	Classification Accuracy (%)
**Prostate cancer**	Liu et al. [28]	22	94.17
	Bir-Jmel et al. [26]	21	**100**
	Sun et al. [18]	4	91.2
	Prabhakar et al. [29]	100	99.48
	Cahyaningrum et al. [30]	10	76.47
	Deng et al. [31]	54	98
	**Proposed**	**3**	**100**

**Table 10:** Summary of statistical significance test result with respect to accuracy between state-of-the-art methods and proposed method.

Dataset	*t*-Value	*p*-Value	Significance Level (0.10)
**Arrhythmia**	−1.618637	0.083225	Significant
**Leukemia**	−2.790643	0.019208	Significant
**DLBCL**	−1.743647	0.070839	Significant
**Prostate cancer**	−1.871934	0.060057	Significant

**Table 11:** Details of three benchmark non-medical datasets.

Dataset	No. of Attributes	No. of Instances	No. of Classes	Dataset Domain
Ionosphere	34	351	2	Electromagnetic
Krvskp	36	3196	2	Game
Sonar	60	208	2	Biology

**Table 12:** Comparison in terms of accuracy (%), and number of selected features obtained by proposed method with some state-of-the-art methods for Ionosphere, Krvskp, and Sonar datasets (highest accuracy and lowest no. of features are highlighted in bold).

Dataset	Method	No. of Features Selected	Classification Accuracy (%)
**Ionosphere**	Ghosh et al. [1]	20	95.36
	Thejas et al. [66]	6	97.18
	Ghosh et al. [2]	7	98.51
	Sheikh et al. [59]	7	**98.56**
	**Proposed**	**5**	95.77
**Krvskp**	Chatterjee et al. [33]	20	97.81
	Sheikh et al. [59]	15	97.81
	Ghosh et al. [34]	11	98.6
	Ghosh et al. [2]	32	**99.06**
	**Proposed**	**4**	95.15
**Sonar**	Ghosh et al. [1]	27	85.07
	Sheikh et al. [59]	22	92.86
	Thejas et al. [66]	51	97.62
	Ghosh et al. [34]	16	**100**
	**Proposed**	**4**	92.85

## Data Availability

We have used only publicly available datasets for experimentation.
